# Occurrence and clinical management of urethral obstruction in male cats under primary veterinary care in the United Kingdom in 2016

**DOI:** 10.1111/jvim.16389

**Published:** 2022-02-24

**Authors:** Dave Beeston, Karen Humm, David B. Church, David Brodbelt, Dan Gerard O'Neill

**Affiliations:** ^1^ Queen Mother Hospital for Animals Royal Veterinary College Hertfordshire United Kingdom; ^2^ Pathobiology and Population Sciences Royal Veterinary College Hertfordshire United Kingdom

**Keywords:** antibiotics, blocked, feline lower urinary tract disease, FLUTD, primary care, recurrence, urinary blockage

## Abstract

**Background:**

Urethral obstruction (UO) has a negative effect on welfare of cats.

**Objectives:**

This study aimed to determine incidence, case management, and outcomes of UO in cats in primary‐care practice in the United Kingdom.

**Animals:**

All male cats under veterinary care within the VetCompass database in 2016.

**Methods:**

A retrospective cohort study was performed. The electronic records of all male cats with a clinical note during the study period were searched for UO cases and were manually reviewed for inclusion. Additional demographic and clinical information were extracted on cases.

**Results:**

From the study cohort of 237 825 male cats, there were 1293 incident cases. The estimated UO incidence risk during 2016 was 0.54 (95% CI: 0.51‐0.57). Demographic and clinical data were available for 1108 cases. Antibiotics were administered to 641/1108 (57.9%) cases. Overall repeat catheterization rate was 253/854 (29.6%). Repeat catheterization at 48 hours was less frequent in patients with indwelling catheters (10.1%) vs those that had a catheter placed and then immediately removed (14.8%; *P* = .04). Death during a UO episode was 329/1108 (29.6%), and 285/329 (88.0%) deaths involved euthanasia.

**Conclusions and Clinical Importance:**

Antibiotics were commonly prescribed in cats for treatment of UO despite minimal evidence in the clinical records of bacterial cystitis. Repeat catheterization was common and case fatality rate during a UO episode was high. Repeat catheterization within 48 hours of elective removal of a urethral catheter was less common in cats that had previously had indwelling catheters. The majority of cats requiring repeat catheterization survived until the end of the study.

**Abbreviations**
EPR: electronic patient record
FLUTD: feline lower urinary tract disease
IQR: interquartile range
ISCAID: International Society for Companion Animal Infectious Disease
rUO: recurrent urethral obstruction
UO: urethral obstruction

## 1 INTRODUCTION

Urethral obstruction (UO) is a common emergency presentation in male cats.^1^ A large retrospective study conducted on male and female cats presenting to veterinary teaching hospitals in the United States and Canada reported an overall incidence of UO of 1.5% over a 19 year period.^2^ The frequency of UO in the wider primary‐care population remains unknown.

UO is reported to predominantly affect young to middle aged (<7 years old) cats which are almost all males because their longer and narrower urethra (compared to females) is more likely to lead to UO.^3^ Causes of UO include urethral plugs consisting of a crystalline matrix, and uroliths, although feline interstitial cystitis is reported in up to 53% of UO cases.^4^ Affected cats present with a nonexpressible bladder and varying levels of cardiovascular stability and azotemia, with particular concern over hyperkalemia‐induced bradyarrhythmia.^5^ Approaches to UO case management discussed in the literature are varied and include both inpatient and outpatient treatment, with limited standardization of treatment options. For example, there are varied reported durations recommended for indwelling catheters, and the use of additional pharmacological aids such as urethral muscle relaxants are controversial.^1^

There is a high risk of UO recurrence (rUO), reported at 11% to 40% of cases^4^, ^6^, ^7^, ^8^; however, comparison between studies is difficult because of variation in treatment methods and patient follow‐up period. Several studies have investigated factors associated with reduced rUO including size and duration of indwelling urethral catheters, use of antibiotics, and additional treatments such as urethral relaxants and antispasmodics.^1^, ^6^, ^7^, ^8^, ^9^ However, factors associated with rUO have not been consistent between studies. For example, 1 study suggested reduced rUO rates with longer catheterization duration,^6^ and another found no difference.^7^

Most published literature on UO in cats is based on information from referral centers, rather than primary‐care practices. This study aimed to estimate incidence of UO diagnosed in cats attending primary‐care practices in the United Kingdom participating in VetCompass, to document the current standards of care applied in these practices and to evaluate survival and recurrence after diagnosis of UO. Finally, we hypothesized that presence of an indwelling urethral catheter is associated with a reduced recurrence rate.

## 2 MATERIALS AND METHODS

A retrospective cohort study was conducted with a sampling frame of all male cats under veterinary care within the VetCompass database for a 1‐year period from January 1, 2016 to December 31, 2016. VetCompass collects and collates anonymized electronic patient records (EPR) from enrolled primary‐care veterinary practices in the United Kingdom.^10^ Patient demographic (species, breed, date of birth, sex, neutering status, bodyweight) and clinical data (free clinical text and treatment fields) are uploaded from veterinary practices to VetCompass for use in research studies.

Cats “under veterinary care” were defined as male cats with at least 1 EPR recorded from January 1 to December 31, 2016. A case of UO was defined as a cat presenting during the study period with partial or total UO and the inability to urinate as recorded in the clinical records. Cats were included as cases if they had a final diagnosis of UO recorded in the EPR for a UO episode first diagnosed in 2016. Cats were excluded if the diagnosis was only presented in a differential list, the diagnosis was later retracted, or the earliest diagnosis of the event was made before January 1, 2016.

Candidate cases of UO were identified using search terms in the clinical notes (obstruct*, urethral, block*, unblock*, u cath*, slippery, indwelling, urin* + cath*) and treatment fields (KatKath, Jackson, Slippery, urin* + cath*) relevant to the diagnosis and management of UO. Search findings were merged and the clinical notes of all candidate cases were examined manually in detail to confirm whether they met the case definition. Demographic data for cases, such as signalment, were extracted automatically from the VetCompass database. Cats were categorized in the follow age groups (years) in alignment with a prior VetCompass study^11^: 0 to <4.5, 4.5 to <9, 9 to <13.5, 13.5 to 18, >18. Nonneutered animals were classified as entire. Further data relating to UO were extracted manually from the EPR. Additional data extracted from the first reported UO episode for incident cases included signalment, date of diagnosis of UO, previous evidence of a UO episode, diagnostics performed on presentation (blood test results, urinalysis, diagnostic imaging), medications dispensed in the first week following diagnosis of UO (analgesics, antibiotics, other), deobstruction attempts and details (whether sedation or anesthesia was used, whether manual expression alone was attempted or whether urethral catheters were placed, catheter type, use of decompressive cystocentesis, indwelling catheter or not), need for repeated catheterization, rUO and time since first diagnosis, transfer of patients to another veterinary practice (other general practice, charity practice, referral practice) and survival. Please note that throughout the manuscript, the term catheterization will refer to urethral catheterization. The urethral catheter composition was determined following data collection to provide a standard set of materials, and to avoid use of brand names. General anesthesia was defined as receiving inhalant anesthetics or notation of “general anesthesia” in the EPR, all other patients receiving pharmacological restraint were classified as sedated. EPRs were checked until their final available record at the time of data collection in April 2020 to June 2020 for evidence of repeat catheterization or mortality. A deobstruction attempt was defined as any attempt at relieving UO and included manual bladder expression and catheterization attempts. A catheterization attempt was defined as any recorded attempt at urethral catheter placement to manage a UO episode and because of inconsistent recording may include multiple catheters, or multiple insertions of the same catheter. Successful catheterization was defined as a recorded catheterization attempt that resulted in voiding of urine and decompression of the bladder. Additional attempts were defined as catheterization attempted under a separate sedation or anesthetic. Death related to UO was defined as evidence of mortality during a UO episode and was further categorized as euthanasia, unassisted or unrecorded.

Data were checked and cleaned in a spreadsheet (Microsoft Office Excel 2016). Cases with evidence of presentation at another practice before the presentation at the current practice for the same UO event were excluded from incidence calculations to prevent possible double counting of records and to allow for greater completeness of data relating to initial assessment and treatment. Cases with evidence of transfer to another practice after initial diagnosis were removed from subsequent analysis of diagnostic and therapeutic management of UO. Incidence described an annual incidence risk and was calculated as the proportion of all male cats under primary veterinary care in 2016 that were diagnosed with UO in the study period. The confidence interval estimates for incidence risk were derived from standard errors, based on approximation to the binomial distribution.^12^ Descriptive statistics were generated for UO cases. Continuous variables were summarized using median, interquartile range (IQR) and range.

Ethical approval for this study was obtained from the Ethics and Welfare Committee at the author's institution for this study (unique reference number: SR2018‐1652).

### 2.1 Statistical analysis

A commercial statistical software program was used for all analyses (SPSS Statistics, version 26; IBM). Binary categorical variables were compared using chi‐square tests. Significance was set at *P* < .05.

## 3 RESULTS

The study cohort comprised of 237 825 male cats under veterinary care at 866 clinics in the United Kingdom during 2016. After review of all 9671 candidate UO cases, 1534 (15.9% of candidates) were confirmed as UO cases. In 241/1534 (15.70%) cases, the EPR documented that the cat had presented to another practice before or during that recorded episode leading to exclusion from analysis, leaving 1293 cases for analysis. The estimated 1‐year incidence risk for UO in male cats was 0.52% (95% CI: 0.49‐0.55). Incidence was highest in cats aged 4.5 years old to 9 years old (0.79%; CI: 0.72‐0.86; Table 1).

**TABLE 1:** Incidence risk of urethral obstruction in cats attending primary care practices in the United Kingdom from January 1, 2016 to December 31, 2016

	All cats (no.)	Incident cases (no.)	Incidence (%) (95% CI^a^)
Overall	237 825	1293	0.54 (0.51‐0.57)
Age (years)			
0 to <4.5	114 011	569	0.50 (0.46‐0.54)
4.5 to < 9	60 361	475	0.79 (0.72–0.86)
9 to <13.5	34 634	174	0.50 (0.43‐0.58)
13.5 to <18	18 681	40	0.21 (0.15‐0.29)
18 to 22.5	4254	4	0.09 (0.03‐0.24)
Breed			
Crossbreed	8386	39	0.47 (0.33‐0.64)
Unrecorded	3038	19	0.63 (0.38‐0.98)
Domestic Short Hair	171 258	1009	0.59 (0.55‐0.63)
Domestic Medium Hair	6135	33	0.54 (0.37‐0.75)
Domestic Long Hair	20 879	111	0.53 (0.44‐0.64)
British Blue	1366	6	0.44 (0.16‐0.95)
Other pure breed	5810	24	0.41 (0.27‐0.61)
Persian	2389	10	0.42 (0.20‐0.77)
Bengal	3309	13	0.39 (0.21‐0.67)
Maine Coon	2284	9	0.39 (0.18‐0.75)
Ragdoll	3728	8	0.21 (0.09–0.42)

*Note*: Data are presented as total number of animlas in the data used in the table unless otherwise stated.

^a^ 95% confidence intervals (CI) are listed.

### 3.1 Descriptive statistics

Median age at diagnosis was 5.0 years (Q1‐Q3: 3.0‐7.5; Min‐Max 0.3‐18.4; age was not recorded in 31 cases). Evidence of a previous UO episode before 2016 was present in 117/1293 (9.0%) of cases.

Electronic patient records documented that cases were transferred to a charity practice in 90/1293 (7.0%) cases, another general practice in 89/1293 (6.9%) cases or referral practice in 6/1293 (0.5%) cases. Cases that were transferred to another practice were not included in further analysis of diagnostics, treatment, and outcome because of a lack of follow up information, leaving 1108 cases for reporting of clinical management.

### 3.2 Diagnostics

Urine for testing was most frequently acquired after catheter placement (537/679 [79.1%]), followed by cystocentesis (85/679 [12.5%]) and free‐catch (34/679 [5%]) and was unrecorded in 23/679 (3.4%) cases that had urinalysis. In‐house urine testing was most common, consisting of a dipstick and specific gravity (506/679 [74.5%]) and/or sediment examination (473/679 [69.7%]). Urine was examined at an external laboratory consisting of dipstick and specific gravity, sediment examination, and culture in 176/679 (25.9%), 185/679 (27.2%) and 217/679 (32%) cases respectively. Blood biochemical profiles were performed in 595/1108 (53.7%) cases, electrolyte analysis in 425/1108 (38.3%) cases and hematology profiles in 183/1108 (16.5%) cases.

Radiography and ultrasonography were performed in 252/1108 (22.8%) and 98/1108 (8.8%) cases, respectively, with both modalities performed in 45/1108 (3.6%) cases. Of the 305 cases that had radiography, ultrasonography or both performed, calculi were identified 31/305 (10.2%) cases. The most common sites for calculi were the bladder (20/31 [64.5%] followed by the urethra (9/31 [29.0%]), kidney (4/31 [12.9%]), and ureter (1/31 [3.2%]).

### 3.3 Therapeutics

A summary of therapeutics administered is found in Table 2. Analgesic administration for management of UO at any point in the first week after diagnosis was recorded in 941/1108 (84.9%) of cases. The most common analgesics dispensed were buprenorphine (743/1108 [67.1%]), meloxicam (729/1108 [65.8%]) and methadone (188/1108 [17.0%]). Other analgesics included tramadol (22/1108 [2.0%]), robenacoxib (11/1108 [1.0%], fentanyl (10/1108 [0.9%]), gabapentin (7/1108 [0.6%]), and carprofen (4/1108 [0.4%]). One cat received a ketamine continuous rate infusion during hospitalization, and 1 cat received an epidural before urethral catheter placement. One hundred sixty‐seven cases (15.1%) were not recorded as receiving any analgesia.

**TABLE 2:** Prescription of antibiotic, analgesic, and additional medications for cats with urethral obstruction presenting to primary care practice in the United Kingdom from January 1, 2016 to December 31, 2016 (N = 1108)

	No.	Percentage (%)
Analgesia		
Buprenorphine	743	84.9
Meloxicam	729	65.8
Methadone	188	17.0
Other	56	5.1
None	167	15.1
Antibiotics		
Amoxicillin‐clavulanic acid	641	57.9
Cefovecin	242	21.8
Enrofloxacin	22	2.0
Other	36	3.2
None	467	42.1
Additional medications		
Prazosin	392	35.4
L‐Tryptophan	337	30.4
Dantrolene	174	15.7
None	445	40.2

Intravenous fluid therapy was administered to 651/1108 (58.8%) of cases. Other therapies administered included calcium gluconate (35/1108 [3.2%]), glucose (15/1108 [1.4%]), neutral “regular” insulin (13/1108 [1.2%]), porcine “long‐acting” insulin (3/1108 [0.3%]), sodium bicarbonate (3/1108 [0.3%]) and lidocaine (1/1108 [0.1%]).

Antibiotics were administered to 641/1108 (57.9%) cases in the first week following UO diagnosis. The antibiotics administered were amoxicillin‐clavulanic acid (405/1108 [36.6%]), cefovecin (242/1108 [21.8%]), enrofloxacin (22/1108 [2%]), cefuroxime (11/1108 [1%]), marbofloxacin (7/1108 [0.6%]), pradofloxacin (6/1108 [0.5%]), cephalexin (5/1108 [0.5%]), metronidazole (3/1108 [0.3%]), doxycycline (2/1108 [0.2%]), benzyl penicillin (1/1108 [0.1%]), and trimethoprim/sulfamethoxazole (1/1108 [0.1%]. Antibiotics were noted to be administered in the EPR but the specific drug was not noted in 2/1108 [0.1%] cases. Of cases receiving antibiotics, 65/641 (10%) received multiple antibiotics, and 150/641 (23.0%) had urine culture performed. Of the 467/1108 (42.1%) cases that did not receive antibiotics, 26/467 (5.6%) had urine culture performed.

Additional medications were prescribed to manage UO in 663/1108 (59.8%) cases. Additional medications were prazosin (392/1108 [35.4%]), L‐tryptophan containing supplement (Cystophan; Protexin; 337/1108 [30.4%]), dantrolene (174/1108 [15.7%]), diazepam (PO, 94/1108 [8.5%]), an alpha‐cazocepine containing supplement (Zylkene; Vetoquinol; 41/1108 [3.7%]), phenoxybenzamine (40/1108 [3.6%]), dexamethasone (29/1108 [2.6%]), a green lipped mussel containing supplement (11/1108 [1.0%]), bethanecol (8/1108 [0.7%]), amitriptyline (5/1108 [0.5%]), furosemide (4/1108 [0.4%]), alprazolam (3/1108 [0.3%]), pentosan polysulfate (2/1108 [0.2%]), and vitamin K [1/1108 [0.1%]).

### 3.4 Catheterization

Deobstruction (relief of the obstruction) was attempted in 938/1108 (84.7%) cases. Of the 170 (15.3%) cases that did not have an attempted deobstruction, 156/170 (91.8%) were euthanized, 11/170 (6.5%) died unassisted, and 3 (1.8%) were lost to follow up. Of the 156 cases that were euthanized, 139 (89.1%) did not have prior evidence of a UO episode.

General anesthesia was the most common form of restraint used for attempted deobstruction, performed in 430/938 (45.8%) cases, followed by sedation (407/938 [43.4%]) and sedation converted to general anesthesia (18/938 [1.9%]. Deobstruction was attempted conscious in 34/938 (3.6%) cases although 8/34 (23.5%) of these cases did require later sedation for successful deobstruction. Method of restraint was not recorded in 49/938 (5.2%) cases.

The most common anesthetic and sedative drugs used to facilitate a deobstruction attempt were buprenorphine (389/938 [41.5%]) propofol (327/938 [34.9%]), medetomidine (316/938 [33.7%]), isoflurane (280/938 [29.9%]), ketamine (227/938 [24.2%]), midazolam (153/938 [16.3%]), butorphanol (145/938 [15.5%]), methadone (122/938 [13%]), acepromazine (115/938 [12.3%]), diazepam (106/938 [11.3%]), dexmedetomidine (36/938 [3.8%]), and sevoflurane (26/938 [2.8%]). The drugs used for sedation, general anesthesia or both were not recorded in 51/938 (5.4%) cases. Of the 887/938 (94.6%) cases where drugs were recorded, 763/887 (86%) cases had more than 1 drug used.

Decompressive cystocentesis was performed before a deobstruction attempt in 127/938 (13.5%) cases. A urethral catheter was used in 867/938 (92.4%) cases where deobstruction was attempted, of which 833/867 (96.1%) were successfully placed on first attempt. Additional attempts at catheterization were successful in 21/34 (61.8%) cases with the remaining 13/34 (38.2%) failing to achieve successful catheterization with additional attempts. Of the 71 cases where sedation, general anesthetic or both was performed and no urethral catheter was used, a combination of decompressive cystocentesis and bladder expression was performed in 53/71 (74.6%) cases, and euthanasia was performed in 18/71 (25.4%) cases.

The most common catheter type used for the first successful catheterization attempt was a polyethylene catheter with stylet (436/854 [51.1%]), followed by a nylon catheter (258/854 [30.2%]), a polytetrafluroethylene catheter (118/854 [13.8%]), a teflon‐based catheter (94/854 [11.0%]), a silicone catheter (60/854 [7.0%]), and a polyurethane catheter (58/854 [6.8%]). The catheter type used was not recorded in 265/854 (31.0%) of EPRs. A catheter was left indwelling in 563/854 (65.9%) cases but was removed before fixation in 291/854 (34.1%) cases. The most common catheter left indwelling was the nylon catheter (100/563 [19.5%]), followed by polytetrafluroethylene catheter (95/563 [16.9%]), polyurethane catheter (61/563 [10.8%]), Teflon‐based catheter (61/563 [10.8%]), silicone catheter (31/563 [5.5%]), and polyethylene catheter with stylet (15/563 [2.7%]). The type of catheter left indwelling was not recorded in 177/563 (31.4%) of EPRs.

Complications relating to an indwelling catheter were reported in 123/563 (21.8%) cases. The most common complications were obstruction of the catheter (51/563 [9.1%]), self‐removal by the cat (45/563 [8.0%]), and urination around the catheter (17/563 [3.0%]).

### 3.5 Outcome

**FIGURE 1:**
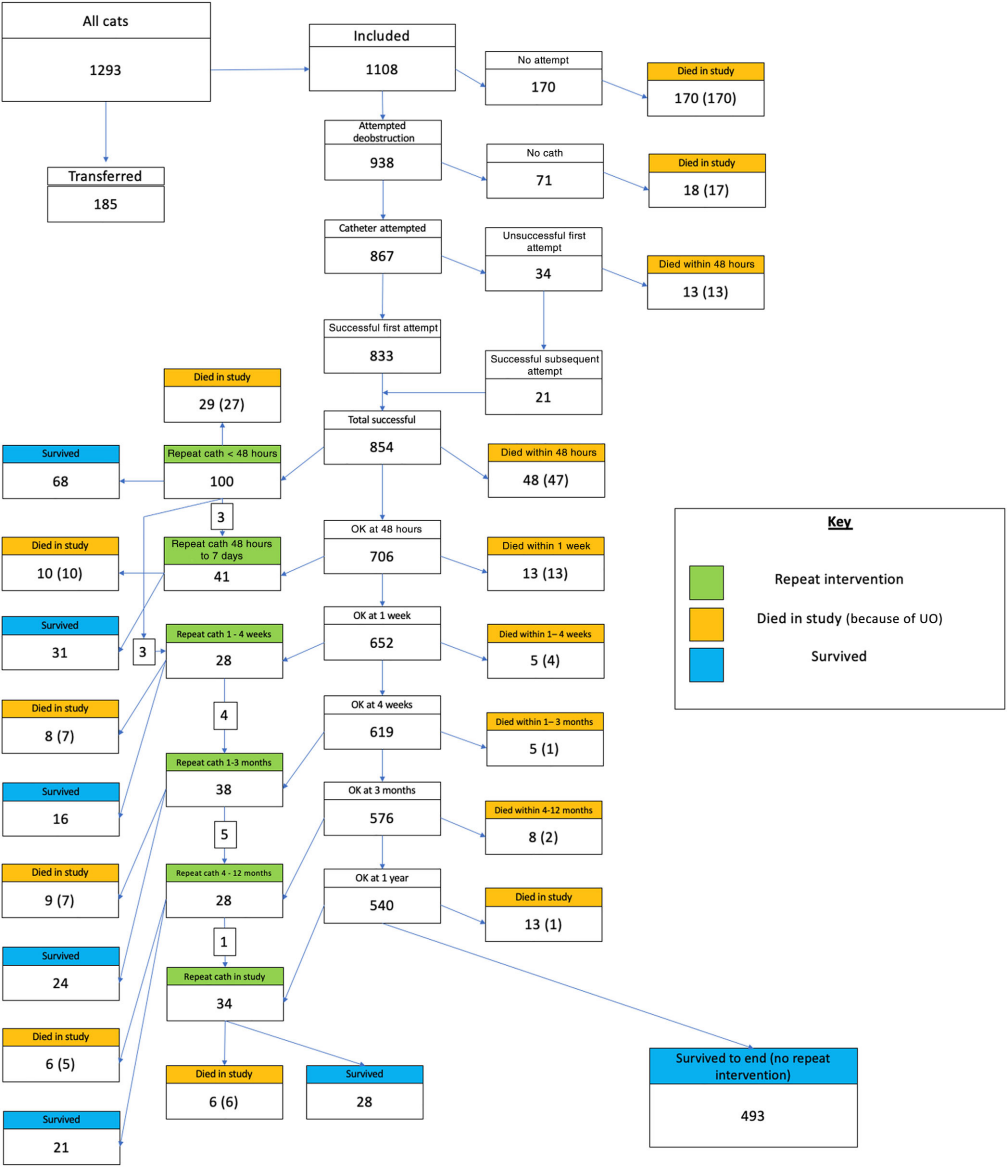
Summary of repeat catheterization, recurrence, and death (euthanasia, unrecorded, and unassisted death) in cats with urethral obstruction presenting to primary care practice in the United Kingdom from January 1, 2016 to December 31, 2016. N = 1108

Outcome and recurrence data are summarized in Figure 1 and Table 3. Repeat catheterization was most common within 48 hours after catheter removal occurring in 100/854 (11.7%) cases, of which 3/100 (3.0%) required further repeat catheterization within 1 week and 3/100 (3.0%) within 1 to 4 weeks. Of 100 cases with repeat catheterization within 48 hours, 68/100 (68.0%) required no further interventions and survived until the end of study, the remaining 32 cats had repeat catheterization (6/32 [18.8%]), repeat catheterization and died (3/32 [9.3%]), or died (25/32 [78.1%]).

**TABLE 3:** Repeat catheterization for cats with urethral obstruction presenting to primary care practice in the United Kingdom from January 1, 2016 to December 31, 2016

Time to repeat catheterization	No.	Percentage
48 h	100/854	11.7%
2–7 d	41/706	5.8%
1–4 wk	28/652	4.3%
1–3 mo	38/619	6.1%
3–12 mo	28/576	4.9%
>12 mo	34/540	6.3%

Of 854 cases with successful urethral catheterization, 493 (57.7%) had no further repeat catheterization and survived until the end of the study. Of the 253 cases that required repeat catheterization at any point in the study, 62/253 (24.5%) were euthanized or died during a UO episode. Repeat catheterization within 48 hours of elective removal of a catheter or discharge from the hospital was significantly more likely in cats in which a catheter was placed and immediately removed (43/291 [14.8%]) compared to cats in which a catheter was left indwelling (57/563 [10.1%]; *P* = .04). However, these cats were not significantly more likely to die during the study period (64/291 died [22.0%] compared to 96/565 with indwelling catheters [17.0%; *P* = .07]).

The overall survival rate to the time of data collection in May 2020 was 67.5% (748/1108). Of the 360 cases that died during the study period, 329/360 (91.4%) died during a UO episode of which 246/329 (74.7%) and 32/329 (9.7%) died within 48 hours and between 2 and 7 days of UO diagnosis, respectively. The remaining 51/329 (15.5%) cats died between 8‐ and 1296‐days post first UO diagnosis. Of the 329 cases that died after a diagnosis of UO, (285/329 [88.0%]) deaths involved euthanasia and the remaining 39/329 (12.0%) died unassisted. The method of death during a UO episode was not recorded in 5/329 (1.5%) cases.

## 4 DISCUSSION

This study reports the occurrence, clinical management strategies, recurrence and survival after diagnosis of UO in male cats attending primary‐care practices in the United Kingdom. Incidence risk of UO in male cats was estimated as 0.52%, with the highest incidence being 0.75% in cats aged 4.5 to 9 years of age.

In the current study, urethral catheterization was attempted in 85% of cats presenting with UO. Only 1% of cases with attempted catheterization were unable to have urethral catheters placed. Catheters were immediately removed in 295/854 (34.5%) cats rather than remaining as indwelling catheters. As the presence of a urethral catheter can cause urethritis,^13^ there has been much debate regarding the optimum duration of retaining indwelling catheters in cats with UO. In our study, cats that had indwelling catheters placed had a lower frequency of repeat catheterization at 48 hours postelective removal of a catheter, or hospital discharge, when compared to those that had the catheter removed after deobstruction; however, there was no significant difference in mortality between groups. A recent prospective cohort study examined the association between standard of care inpatient treatment (defined as an indwelling catheter for at least 12 hours) and, when declined, outpatient treatment with a urethral catheter being placed and then immediately removed, and found an odds ratio of 3 : 1 for increased risk of recurrence of UO in the outpatient group.^8^ These findings, in conjunction with our data, support the use of indwelling catheters to reduce rUO rates. However, repeat catheterization rates for cases in our study with in‐out catheterization were lower than previously reported,^8^ and so might provide an alternative to euthanasia when client finances do not permit hospitalization.

In our study, urinalysis was the most frequently performed diagnostic test; however, only 591/1108 (53.3%) cats had a sediment exam performed. Urethral plugs are reported as a common cause of UO in cats.^4^ Most urethral plugs consist of large quantities of mucoprotein and inflammatory debris which trap mineral crystals.^14^ Of 618 urethral plugs submitted to the Canadian Veterinary Urolith Centre, only 10% of plugs did not contain mineral crystals.^14^ Given that crystalluria can contribute to formation of uroliths and mucus plugs, can be readily identified on urine sediment examination, and can possibly be addressed with dietary manipulation,^15^ urinalysis may play an important part in the diagnostic work up of cats with UO. Imaging was performed rarely in our study, with only 305/1108 (27.1%) cases undergoing radiography, ultrasound, or a combination of both. Urolithiasis has a prevalence of 29% to 47% in cats with UO and can be a treatable cause of rUO.^4^, ^16^ Urolithiasis was radiographically or ultrasonographically identified in only 10.2% of cats that underwent diagnostic imaging in our study, and could suggest a decline in the number of cats suffering from urolithiasis; however, this finding is limited by the retrospective nature of our study. Further prospective studies are warranted to investigate this finding and evaluate 386 the utility of diagnostic imaging in male cats with UO.

Antibiotics were administered to 641/1108 (57.9%) cases as part of the treatment of UO and 150/641 (23.4%) had urine culture performed. While a retrospective study found that 44% of cats with UO had positive urine bacterial culture, urine was obtained from the indwelling urethral catheter in several cases and also from cats with recent previous catheterization.^17^ A recent prospective study of cats managed with indwelling catheters for UO found that no cats had positive cystocentesis urine cultures at the time of admittance before urethral catheterization, with 4/31 (13%) developing positive cultures during hospitalization.^18^ Given the absence of positive urine cultures at the time of urethral catheter placement in that study, it seems that urine culture provides little useful additional information in cats without recent catheterization and might be lower priority in cases with financial constraints. The lack of positive urine cultures also suggests that antibiotics are not routinely indicated in cases of UO.^18^ Currently, the International Society for Companion Animal Infectious Disease (ISCAID) recommends that antibiotics should not be routinely used for cats with indwelling urethral catheters either during placement, or after removal, because of the increased risk associated with antimicrobial‐resistant infections in patients with indwelling devices.^19^ Instead, cystocentesis and urine culture of cats that develop persistent cystitis signs after removal of a urethral catheter is recommended.^19^

Analgesia was administered to 85% of cases as part of the treatment of UO. Urethral obstruction is thought to be a painful condition because of bladder distension and underlying urinary tract pathology and so administration of analgesia should form an early part of any management strategy to reduce pain and requirements for anesthetic drugs used for deobstruction. The 2 most commonly administered analgesic medications in our study were buprenorphine and meloxicam. Cats with interstitial cystitis have evidence of chronic inflammation and remodeling of the urinary bladder that has pathophysiological changes similar to nonulcerative interstitial cystitis/bladder pain syndrome (IC/BPS) in people.^20^ Meloxicam is a nonsteroidal anti‐inflammatory drug (NSAID) that has a theoretical physiological benefit in cats with FLUTD; however, 2 small prospective studies assessing meloxicam use have failed to demonstrate a significant improvement in clinical signs^21^, ^22^ and NSAIDs are a risk factor for worsening acute kidney injury (AKI) in acutely azotemic patients so might best be avoided in UO patients. NSAIDs are not recommended for use in IC/BPS in people, and the American Urological Association suggest a multimodal approach including behavior modification, physiotherapy and nonanalgesic pharmacologic manipulation including amitriptyline and pentosan polysulfate.^23^ Similar recommendations have been recommended in cats for the treatment of interstitial cystitis.^24^ Buprenorphine is a partial agonist at the μ‐opioid receptor that has been extensively reviewed for use in cats^25^; however, its use in FLUTD has not been evaluated. Given the improved safety profile of buprenorphine when compared to meloxicam, it may be prudent to consider preferential use of buprenorphine in cats with UO. Consideration should also be given to other analgesic interventions in acute management of UO such as locoregional anesthesia, other opioids (eg, methadone and fentanyl), and gabapentin.

Cats with UO can present along a spectrum of hemodynamic instability because of potential for life‐threatening hyperkalemia.^5^ Calcium gluconate, insulin, and sodium bicarbonate were used infrequently in cats in this study, with only 3%, 1%, and 0.3% receiving them, respectively. In the current EPR format, we are unable to say whether this represents a lack of requirement for these therapies because cats were less severely affected, whether they were given but not recorded, or whether primary care practices were not evaluating these cases for the presence of hyperkalemia.

An variety of therapeutics aimed to cause urethral relaxation or reduce urinary tract inflammation were administered to the UO cases in the current study. Currently, there is little evidence for or against the use of any of the medications described for feline UO management. The most commonly administered drug in this study was prazosin, an α1‐adrenergic antagonist which causes smooth muscle relaxation. The proximal third of the feline urethra is comprised of smooth muscle with the remaining distal tissue comprising of skeletal muscle^26^ where α‐1‐adrenergic antagonists will have no effect. The majority of obstructions are thought to occur in the distal urethra^27^ and despite the frequent use of prazosin in cats with UO, there is little evidence to suggest any benefit. Although a retrospective study showed reduced risk of rUO in cats treated with prazosin compared to cats receiving phenoxybenzamine,^7^ a recent double‐blinded, prospective, interventional study did not identify a difference in the rate of rUO in cats receiving prazosin, although this study was underpowered to detect a difference.^9^ Hypotension is an adverse effect of prazosin, so it isrecommended to monitor blood pressure during treatment.^9^ L‐Tryptophan containing supplements were frequently prescribed in this study. Tryptophan is an essential amino acid and precursor to both melatonin and serotonin which has been implicated in the susceptibility of cats to stress.^28^ In a recent study there was a reduced rate of rUO of LUTD signs in cats receiving a therapeutic urinary stress diet; however, none of these cats had UO.^29^ It is thought that stress could contribute to the pathophysiology of FLUTD and development of an inflammatory cystitis; however, only 1 study has found a direct association between stress and urinary bladder changes.^30^ Interestingly, diazepam was administered orally in 94/1108 (8.5%) cases. Diazepam (PO) is associated with idiosyncratic acute hepatic necrosis in cats^31^, ^32^ and has no effect on distal urethral pressures in experimental studies when administered IV,^33^ suggesting little benefit, and possible harm, associated with its use in these cats.

In the current study, cats that had repeat catheterization were most frequently recatheterized within 48 hours of elective removal of the catheter or discharge from hospital. This finding is similar to that of a large retrospective study which found the majority of repeat catheterization occurred within the first 4 days postcatheter removal.^7^ Despite a need for repeat catheterization within 48 hours of placement of the first catheter in 100 cases in the current study, 68/100 (68.0%) required no further catheterization and survived to the end of the study. Of the 32 remaining cats, 28/32 (87.5%) died during the study period. There are varying rates of rUO reported in the literature; however, direct comparisons are difficult because of different outcome measures, for example, short and long‐term recurrence vs overall recurrence, and different durations of observation. Several studies have looked at rUO at 30 days, with reported recurrence rates of 10.9% to 14.7%^6^, ^7^ which is slightly lower than the total 28 day repeat catheterization rate in our study (19.7%). Overall repeat catheterization rate was 29.6% in our study, with 25.8% of cases presenting within the first year. Long term rUO has been assessed in 2 previous studies with rates of 24% and 36% for 2 and 3 years, respectively.^4^, ^34^ As patient records were monitored to the time of data collection, our study period covered more than a 3‐year period for many cats, suggesting our rUO rate was similar to previous literature.

Our study identified that 29.6% of the cases did not survive the initial UO event. Urethral obstruction has previously been reported with a case fatality rate of 6.4% to 26.0%.^4^, ^5^, ^17^ The variation in reported case fatalityrate is likely impacted by the heterogeneity of follow‐up duration and the type of clinic to which the cat presents to (referral vs primary‐care). As seen in Figure 1, the majority of cases that did not survive died within the first 48 hours, accounting for 62.7% of all UO‐related deaths. Similar to other published literature, the most common method of death in our study was euthanasia which occurred in 86.7% of deaths. However, inferences on the justification for the euthanasia decision cannot be made from this study as full information detailing the precise clinical reasoning were often not recorded. One hundred thirty‐nine cats (12.5%) with no evidence of prior UO were euthanized without attempted catheterization, suggesting owners may have had marked welfare or financial concerns, or that veterinary recommendations have been made based on assumption of poor long‐term prognosis. Of cats with successful catheterization, 493/854 (57.7%) had no further catheterization and survived until the end of study. Of cats that required more than 1 catheterization, 188/253 (74.3%) survived until the end of study.

There were several limitations in this study. The EPR data were not originally recorded for research purposes, so information may have been inconsistently recorded. This prevented more detailed evaluation of parameters, for example duration of indwelling catheters before removal or size of catheter, or for assessment of electrocardiogram abnormalities and presence of hyperkalemia. As such, it is not possible to conclude whether absence of recorded details could be safely taken to confirm true absence. One example is the low levels of recording of chronic management strategies such as multimodal environmental modification (MEMO). MEMO has been associated with reduced recurrence of nonobstructive FLUTD^24^; however, we were unable to assess its implementation in our study because of inconsistent EPR documentation. Similarly, biochemistry, urinalysis and culture results were infrequently documented, limiting their inclusion. EPRs were evaluated until the last recorded entry; however, cats that moved to another veterinary practice after their UO may have been lost to follow up and therefore the true longer‐term rUO and case fatality rates maight be underestimated. Insurance and financial data were not available for this study. Demographic data such as neuter status was not manually checked in the EPR which could explain our large number of entire animals. It is possible that our high case fatality rate was influenced by financial constraints, or by recommendations based on previous literature of high rUO rates. Because of the higher case fatality rate demonstrated in this study, it is possible that repeat catheterization rates were artificially lowered by patients being euthanized.

## 5 CONCLUSIONS

Urethral obstruction is seen in 1‐in‐200 male cats under primary veterinary care each year in the United Kingdom and is associated with high euthanasia rates. Euthanasia was performed during a UO episode in just over 1 in 4 cats presenting to primary‐care practices in the United Kingdom. A relatively low number of cats undergo important diagnostics such as urine sediment examination and imaging. Many cats receive additional treatments such as antibiotics or muscle relaxants despite limited evidence supporting their use and potential for harm. Repeated catheterization is common, occurring in 11.7% cases within the first 48 hours after initial presentation and in 29.6% cases overall. Despite frequent recatheterization in the first 48 hours, many of these cats do not have subsequent rUO. Cats with indwelling catheters were significantly less likely to have repeat catheterization at 48 hours postremoval compared to those without indwelling catheters.

## CONFLICT OF INTEREST DECLARATION

Authors declare no conflict of interest.

## OFF‐LABEL ANTIMICROBIAL DECLARATION

Authors declare no off‐label use of antimicrobials.

## INSTITUTIONAL ANIMAL CARE AND USE COMMITTEE (IACUC) OR OTHER APPROVAL DECLARATION

Approved by the Royal Veterinary College Ethics and Welfare Committee, reference number SR2018‐1652.

## HUMAN ETHICS APPROVAL DECLARATION

Authors declare human ethics approval was not needed for this study.
